# Impact of the World Health Organization Pain Treatment Guidelines and the European Medicines Agency Safety Recommendations on Nonsteroidal Anti-Inflammatory Drug Use in Lithuania: An Observational Study

**DOI:** 10.3390/medicina54020030

**Published:** 2018-05-11

**Authors:** Skaistė Kasciuškevičiūtė, Gintautas Gumbrevičius, Aušra Vendzelytė, Arūnas Ščiupokas, Kęstutis Petrikonis, Edmundas Kaduševičius

**Affiliations:** 1 Division of Clinical Pharmacology, Institute of Physiology and Pharmacology, Medical Academy, Lithuanian University of Health Sciences, 44307 Kaunas, Lithuania; gingum59@gmail.com (G.G.); edmundas.kadusevicius@lsmuni.lt (E.K.); 2 Faculty of Pharmacy, Medical Academy, Lithuanian University of Health Sciences, 44307 Kaunas, Lithuania; a.vendzelyte@gmail.com; 3 Department of Neurology, Medical Academy, Lithuanian University of Health Sciences, 44307 Kaunas, Lithuania; asciupokas@hotmail.com (A.S.); kestutispetrikonis@yahoo.com (K.P.)

**Keywords:** rational prescribing, analgesic, guidelines, NSAID, utilization

## Abstract

*Background and objective:* Irrational use of nonsteroidal anti-inflammatory drugs (NSAIDs) is the main cause of adverse effects-associated hospitalizations among all medication groups leading to extremely increased costs for health care. Pharmacoepidemiological studies can partly reveal such issues and encourage further decisions. Therefore, the aim of our study was to evaluate the utilization of non-opioid analgesics (ATC classification N02B and M01A) in Lithuania, and to compare it with that of other Baltic and Scandinavian countries in terms of compliance to the WHO pain treatment guidelines and the EMA safety recommendations on NSAID use. *Materials and methods:* The dispensing data were obtained from the sales analysis software provider in the Baltic countries (SoftDent, Ltd., Kaunas, Lithuania); State Medicine Control Agencies of Lithuania, Latvia, and Estonia; Norwegian Prescription Database; Swedish Database for Medicines; and Danish Prescription Database. Data included the utilization of both prescription and over-the-counter drugs. Utilization was expressed in defined daily doses (DDD)/1000 inhabitants/day. *Results:* During the 11-year period, the utilization of drugs belonging to the N02B and M01A groups increased by 22.8%, from 58.37 in 2005 to 71.68 DDD/1000 inhabitants/day in 2016 in Lithuania. Contrary to the WHO guidelines on pain management, all Baltic countries were more likely to use NSAIDs than other analgesics and antipyretics: in 2015, the drugs of the M01A group were used 6.04, 5.79, and 6.11 times more than those of N02B in Lithuania, Estonia, and Latvia, respectively, whereas the Scandinavian countries preferred the N02B to the M01A group: in Denmark and Sweden, the utilization of other analgesics and antipyretics was 2.33 and 1.24, respectively, times higher than that of NSAIDs. In Norway, the use of both groups was similar. In the Scandinavian countries, paracetamol was the analgesic of first choice, whereas, in Lithuania, it took only the third place. The most popular drug in Lithuania was diclofenac, and its utilization accounted for 30.04% of all non-opioid analgesics in 2016. Although the European Medicines Agency (EMA) restricted the use of certain NSAIDs, i.e., cyclooxygenase-2 (COX-2) inhibitors, nimesulide, and diclofenac, their use consistently increased by 15.91, 2.83, and 1.41 times, respectively, showing incompliance with the international guidelines. *Conclusions:* Neither the EMA safety policy on NSAID use nor the WHO pain treatment guidelines had a sufficient impact on the rational use of NSAIDs in Lithuania. The use of NSAIDs restricted by the EMA (diclofenac, COX-2 inhibitors, nimesulide, and piroxicam) remains high or even increases, while the utilization of safer alternatives (paracetamol and naproxen) remains relatively low as compared with the Scandinavian countries. Incompliance with international guidelines may result in increased morbidity, mortality and higher costs for health care.

## 1. Introduction

Nonsteroidal anti-inflammatory drugs (NSAIDs) are one of the most commonly used classes of drugs in the world—it is reported that about 90% of the analgesics used belong to NSAIDs [1,2]. More than 30 million people worldwide use NSAIDs every day; more than 111 million prescriptions for NSAIDs are dispensed in the United States of America (USA) annually, which accounts for approximately 60% of the USA over-the-counter analgesic market [3]. Moreover, recent studies have shown that the overall utilization of analgesics increased considerably over the last decades, although there are substantial differences in trends toward utilization of particular analgesics among countries [4,5,6,7,8].

While NSAIDs are the most widely used drugs, especially among the elderly who frequently use them on a long-term basis, the benefit–risk balance of individual NSAIDs is chiefly driven by their gastrointestinal (GI) and cardiovascular (CV) safety profile. A systematic literature review revealed that NSAIDs are among the ten top groups of medications/medication associated with the most serious medication errors, i.e., the errors that cause damage or pose a threat of harm to a patient: NSAIDs accounted for 4% of all the drugs causing fatal events and 8% of the drugs causing hospitalizations, prolonged hospital stay, life-threatening conditions, and disability due to medication errors [9]. NSAID-associated gastropathy accounts for at least 165,000 deaths and 650,000 hospitalizations each year worldwide. The number of deaths associated with NSAIDs is similar to that from acquired immunodeficiency syndrome and considerably greater than the number of deaths from multiple myeloma, asthma, cervical cancer, or Hodgkin’s disease [10]. To lower the associated risk of GI side effects, selective cyclooxygenase-2 inhibitors were introduced as anti-inflammatory drugs of a new generation. However, the use of COX-2 inhibitors was also found to be associated with significant CV risk in the VIGOR (Vioxx GI Outcomes research) study in 2000: rofecoxib users had a higher prevalence of myocardial infarction. In 2004, the Adenomatous Polyp Prevention on Vioxx (APPROVe) clinical trial confirmed the CV complications associated with rofecoxib, resulting in the withdrawal of rofecoxib in 2004 [11,12,13,14,15]. Since then, several studies have confirmed the unfavorable CV safety profile of COX-2 inhibitors, and later it has been extended to traditional (nonselective) NSAIDs as well [12,16,17,18,19,20,21]. The meta-analysis performed by the Coxib and traditional NSAID Trialists’ (CNT) Collaboration has revealed that coxibs, diclofenac, and ibuprofen increased the risk of major vascular events by approximately 40%, the risk of hospitalization due to heart failure was roughly doubled by all coxibs and traditional NSAID regimens studied, and the risk of vascular death was significantly increased by coxibs and diclofenac [22]. Following the above-mentioned studies confirming an unfavorable CV safety profile associated with coxibs and some traditional NSAIDs, the European Medicine Agency (EMA) has published the recommendations on the safety precautions of particular NSAIDs, which should serve as clinical practice guidelines helping to choose the most appropriate analgesic in pain management [22,23,24,25,26].

Overall, clinical guidelines are an important tool helping to rationalize any disease management and resulting in more effective, less costly clinical outcomes and fewer complications or treatment-associated side effects [27,28,29,30,31,32]. As a result, EMA recommendations on the safety precautions of NSAIDs, which are based on meta-analyses and randomized clinical trials, should play a key role in pain management, particularly considering that irrational NSAID use is associated with potential hazardous and life-threatening outcomes as mentioned above. Hence, this study aimed to evaluate trends in the utilization of NSAIDs and other analgesics (M01A and N02B pharmacological groups) over the last 11 years in Lithuania, comparing it with other European countries, and to reveal compliance with the international pain management guidelines in real-life clinical practice. It is assumed that the results of this study could help rationalize the use of analgesics.

## 2. Materials and Sources

### 2.1. Data Sources

The dispensing data of non-opioid analgesics were obtained from the sales analysis software provider in the Baltic countries (SoftDent, Ltd.); State Medicine Control Agencies of Lithuania, Latvia and Estonia; Norwegian Prescription Database; Swedish Database for Medicines; and Danish Prescription Database. Data included the utilization of both prescription and over-the-counter drugs [33,34,35,36]. The main limitation of these databases is that they provide measures of opioid utilization using dispensing claims and, therefore, do not provide a direct measure of medicine consumption.

### 2.2. Measures of Analgesics Utilization

The Anatomical Therapeutic Chemical/Defined Daily Dose (ATC/DDD) methodology was used to analyze analgesic utilization. Analgesic utilization was measured as the number of DDDs and expressed as number of DDDs per 1000 inhabitants per day and their percentage. The overall utilization was calculated for the total population including all age groups. The N02B and M01A groups according to the WHO/ATC classification were included. A total of 4 N02B (paracetamol, metamizole natrium, flupirtine, and aspirin) and 20 M01A (ibuprofen, diclofenac, and other NSAIDs) medicines available on the market were evaluated, i.e., the drug utilization volume was 100% (DU 100%). To provide a summary of data, descriptive statistics were used.

## 3. Results

### 3.1. Utilization Analysis of Pain and Inflammation Relievers (N02B and M01A Pharmacotherapeutic Groups) in Lithuania over the 11-Year Period (2005–2016)

**Figure 1:**
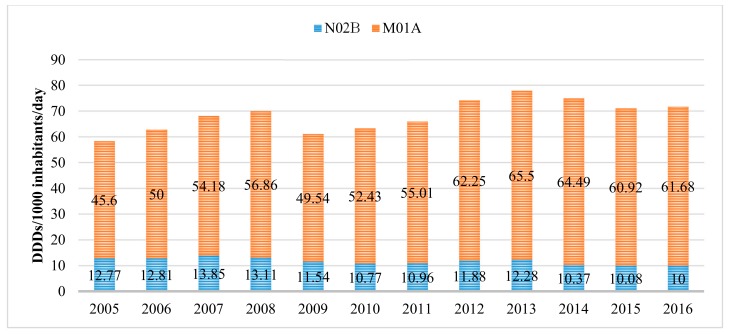
Utilization of non-opioid analgesics (ATC codes N02B and M01A) (expressed as DDDs/10,000 inhabitants/day) in Lithuania during 2005–2016. NSAIDs were much more popular than other non-opioid analgesics.

Over the 11-year period, the overall utilization of pain and inflammation relievers (N02B and M01A pharmacotherapeutic groups) increased by 22.8%, i.e., from 58.37 DDD/1000 inhabitants/day in 2005 to 71.68 DDD/1000 inhabitants/day in 2016. Such an increase was documented exclusively due to a significant rise (by 35.26%) in the use of NSAIDs (M01A), while the utilization of other analgesics (N02B) even decreased. Overall, the use of NSAIDs was 6.17 times higher than that of other analgesics and antipyretics (N02B) in 2016. The detailed utilization data are provided in Figure 1.

**Figure 2:**
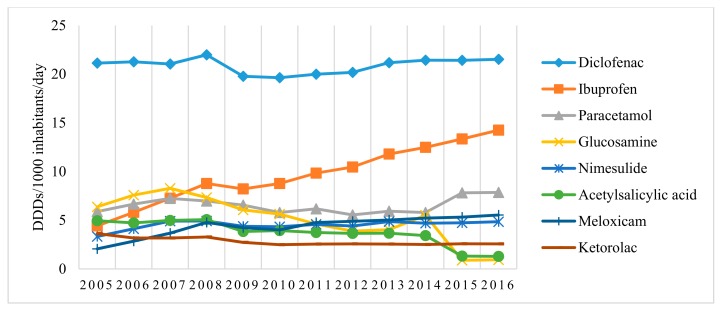
Trends in the use of the most popular analgesics in Lithuania during 2005–2016.

Diclofenac was the most commonly used medication of the M01A group; besides, its utilization remained constant during the study period and accounted for nearly one-third (30.04% in 2016) of all analgesics (including M01A and N02B) used. Ibuprofen, meloxicam, and nimesulide also showed a continuous increase in utilization during the study period: the utilization of ibuprofen increased by 3.22 time, from 4.421 in 2005 to 14.252 DDD/1000 inhabitants/day in 2016; meloxicam, by 169.2% from 2.058 in 2005 to 5.54 DDD/1000 inhabitants/day in 2016; and nimesulide, by 45.6% from 3.332 in 2005 to 4.851 DDD/1000 inhabitants/day in 2016 (Figure 2).

**Figure 3:**
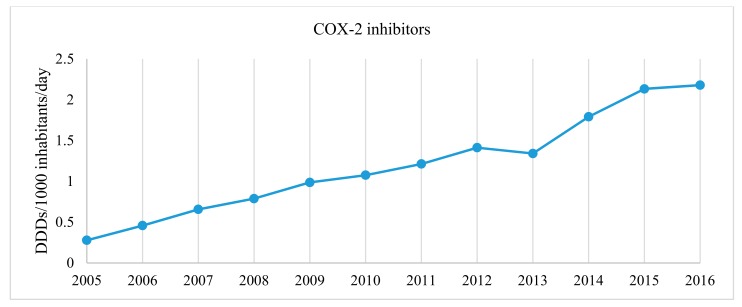
Trends in the utilization of COX-2 inhibitors in Lithuania from 2005 to 2016.

However, the most prominent changes were recorded in the utilization of selective COX-2 inhibitors (celecoxib and etoricoxib): their use increased even 7.81 times from 0.279 in 2005 to 2.178 DDD/1000 inhabitants/day in 2016, indicating different trends than could be influenced by the EMA restrictions on the use of COX-2 inhibitors approved in 2005 (Figure 3). On the other hand, some drugs in M01A group exhibited a continuous decrease in utilization, i.e., glucosamine and ketorolac utilization gradually decreased by 24.76% and 23.52%, respectively (Figure 2).

As far as the N02B pharmaceutical group (paracetamol, acetylsalicylic acid, metamizole sodium, and flupirtine) is concerned, an overall decrease in utilization (−21.68%) was observed. Trends in the use of the plain N02B medicines as well as combinations with other active ingredients such as caffeine or fixed-dose combinations of two N02B medicines were analyzed. Although paracetamol was the most commonly used medication of the N02B group, according to its utilization, paracetamol took only the third place among all the studied medications. In 2005, the utilization of total paracetamol (either alone or in combination with other medicinal substances), plain paracetamol, its combinations with other medicinal substances (e.g., codeine, caffeine, etc.) was 5.883, 2.951, and 2.932 DDD/1000 inhabitants/day, respectively, whereas, in 2016, 7.853, 3.031, and 4.822 DDD/1000 inhabitants/day, respectively. Therefore, the total use of paracetamol (both plain and combination products) increased by 33.49%, mainly due to a significant rise in the use of paracetamol-containing combination products—from 2.933 to 4.822 DDD/1000 inhabitants/day. Similarly, the utilization of acetylsalicylic acid in combination with other substances such as caffeine, paracetamol, and others was 2.1 times higher than that of plain acetylsalicylic acid (30.009 and 14.59 DDD/1000 inhabitants/day, respectively) over the 2005–2016 period. Of note, the use of acetylsalicylic acid including medicines in combinations decreased markedly (by 73.93%) during the study period: from 4.953 in 2005 to 1.291 DDD/1000 inhabitants/day in 2016.

### 3.2. Comparison of Analgesic Utilization in Lithuania and Other Baltic and Scandinavian Countries

**Figure 4:**
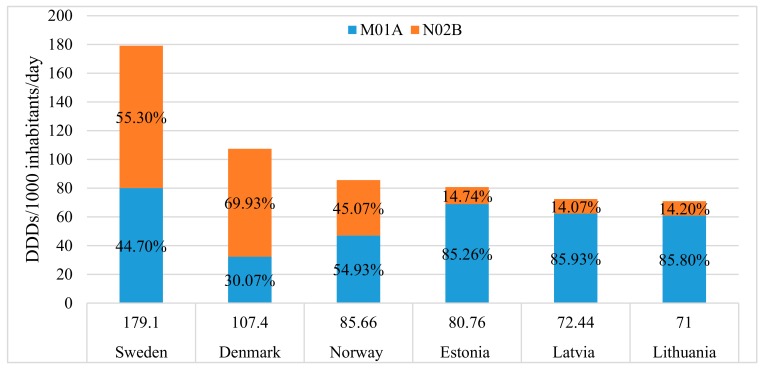
The utilization of non-opioid analgesics and proportions of M01A and N02B pharmacotherapeutic groups in the Baltic and Scandinavian countries, 2015.

The overall utilization of nonsteroidal anti-inflammatory and anti-rheumatic medicinal products and other analgesics and antipyretics (ATC codes M01A and N02B) was 71 DDD/1000 inhabitants/day in Lithuania in 2015. Compared with other Baltic and Scandinavian countries, Lithuania showed similar indicators of utilization to other Baltic countries, but significantly lower than in Sweden, Denmark, and Norway (Figure 4), indicating that in general Lithuania remains a country with a relatively low level of analgesic utilization.

In addition, this study highlighted the differences in the preferred group of analgesics between the Scandinavian and the Baltic countries. The Baltic countries were more likely to use NSAIDs than other analgesics and antipyretics: in 2015, the drugs belonging to the M01A group were consumed 6.04, 5.79, and 6.11 times more than those of N02B in Lithuania, Estonia, and Latvia, respectively. On the contrary, Scandinavian countries preferred the N02B to the M01A group: in Denmark and Sweden, the utilization of other analgesics and antipyretics was 2.33 and 1.24, respectively, times higher than that of NSAIDs. In Norway, the use of both groups was similar (Figure 4).

**Figure 5:**
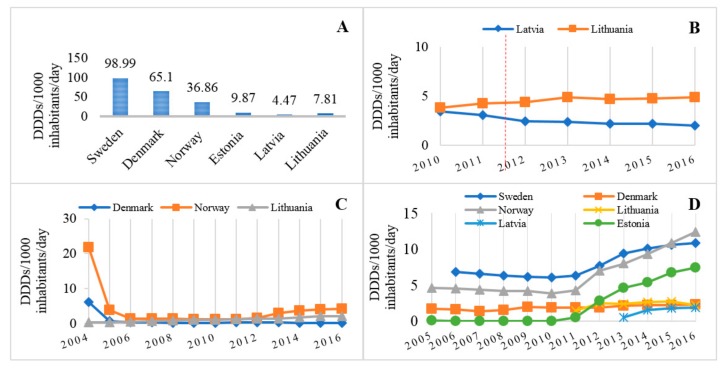
The utilization of non-opioid analgesics in Lithuania compared to other Baltic and Scandinavian countries: (**A**) the utilization of paracetamol in 2015; (**B**) trends in nimesulide use in Lithuania and Latvia (vertical dashed line indicates the time when the EMA enhanced restrictions on nimesulide use due to concerns of hepatotoxicity) (note: nimesulide is not authorized in Estonia, Denmark, Norway and Sweden); (**C**) trends in COX-2 inhibitor utilization after rofecoxib and valdecoxib withdrawal from the market in 2004 (note: data for years 2004–2005 were unavailable for Sweden, Latvia, and Estonia); and (**D**) trends in naproxen utilization in 2005–2016.

Lithuania, Sweden, and Latvia were the leading countries according to the utilization of diclofenac (21.44, 16.47, and 22.56 DDD/1000 inhabitants/day, respectively), while it was not so commonly used in Norway, Estonia, and Denmark (8.86, 11.17, and 2.9 DDD/1000 inhabitants/day, respectively). Interestingly, the utilization of paracetamol in Lithuania accounted only for 11% of all analgesics (both M01A and N02B) used in 2015, while, in Sweden, Denmark, and Norway, this percentage was 55.27%, 60.61%, and 43.03%, respectively [36,37,38,39]. Paracetamol utilization in Lithuania and other Baltic and Scandinavian countries is shown in Figure 5A.

The use of nimesulide continued to increase in Lithuania, even after 2012, when the EMA enhanced the previously approved restrictions on its use because of the potential risk of hepatotoxicity (the use increased by 10% over this period), while, in Latvia, the use of nimesulide continued to decrease (Figure 5B).

Similar trends were observed for the utilization of COX-2 inhibitors: their use decreased 5 times in Norway and even 31 times in Denmark after clinical trials revealed the potential CV risk, while, in Lithuania, their use consistently continued to increase. Trends in the utilization of COX-2 inhibitors following the worldwide withdrawal of rofecoxib from the market is shown in Figure 5C. Unfortunately, data prior to 2004 are unavailable for all these countries, and complete assessment of changes could not be made.

Since 2005, the utilization of naproxen has significantly increased in Sweden and Denmark (by 1.6 and 2.7 times, respectively). In Lithuania, naproxen was introduced into the market only in 2011, and its utilization increased 1.7 times during the first marketing year; however, later it was stable (around 2.2–2.7 DDD/1000 inhabitants/day) and remained one of the least used NSAID during the study period. Trends in naproxen utilization are displayed in Figure 5D.

## 4. Discussion

### 4.1. General Considerations

All NSAIDs have equivalent efficacy but different safety profile. Traditional NSAIDs are associated with an increased risk of serious GI adverse effects, while selective COX-2 inhibitors were introduced as NSAIDs of a new generation, specifically designed to lower the risk of GI side effects [37,38,39,40,41,42]. However, further studies have revealed an increased risk of CV events associated with COX-2 inhibitors, followed by rofecoxib and valdecoxib withdrawal from the market [11,21,43,44]. On the other hand, recent studies and meta-analyses have shown that all NSAIDs (non-selective, selective COX-2 inhibitors) might cause adverse CV effects to a problematic extent, with naproxen and low-dose ibuprofen posing the lowest risk of [21,45,46,47]. All this indicates the complexity of NSAID safety profile, suggesting that the clinical decision which drug to choose should be based on detailed evaluation of the risk of CV effects and GI complications as well as the other common adverse effects such as renovascular, hepatotoxic, or phototoxic. To optimize the choice of a particular NSAID and prevent potential harm associated with an irrational NSAID use, a clinician should guide evidence-based recommendations. The most widely used and well-known recommendations—EMA safety recommendations and the WHO analgesic ladder—and compliance with them are discussed below.

### 4.2. Pain Management Compliance with WHO Pain Treatment Guidelines

In 1986, the WHO introduced the analgesic ladder as a framework that physicians could use when making decisions for cancer pain management; later it was extended also to non-malignant chronic pain and served as the first international pain management guidelines. The WHO analgesic ladder provides a three-step sequential approach for analgesic administration based on pain severity and has global applicability [48]. These therapeutic guidelines paved the way for considerable improvement in the management of chronic pain, and after 30 years of use, the analgesic ladder has demonstrated its effectiveness and widespread usefulness [49]. In reference to the WHO pain relief ladder as well as many other international guidelines and recommendations, paracetamol is a drug of first choice for relieving mild to moderate pain especially due to its favorable safety profile [48,50,51,52,53,54,55]. Such recommendations arise from a favorable safety profile of paracetamol compared to that of NSAIDs—paracetamol is considered safer in terms of gastrointestinal, cardiovascular, renal, and hepatic adverse effects [56,57,58,59]. As a result, paracetamol should be the leading non-opioid analgesic in terms of the utilization levels. A good example demonstrating that paracetamol could be used as a first-line analgesic is Scandinavian countries, where paracetamol is the most commonly used non-opioid analgesic. Unfortunately, in Lithuania, the role of paracetamol as an analgesic seems to be underestimated resulting in only the third place by utilization as compared to other non-opioid analgesics. Relatively low utilization of paracetamol is one of the major negative finding of this observational study indirectly showing that the WHO recommendations for pain management are not influential enough to guide the decision which analgesic to choose.

### 4.3. Pain Management Compliance with Recommendations of the European Medicines Agency

The EMA is the principal regulatory authority in the European Union, which constantly evaluates and regularly updates information on the balance of benefits and risk of medicines. Consequent recommendations are considered mandatory in all Member States and should serve as guidelines for relevant decision making. Since 2005, the EMA has restricted the use of certain NSAIDs due to concerns about safety.

In 2005, the EMA Committee for Medicinal Products for Human Use (CHMP) restricted the use of COX-2 inhibitors taking into account the results of the above-mentioned studies, showing the potential CV risk. Following these precautions, the utilization of coxibs decreased extremely in Denmark and Norway. Studies in other countries also showed that the use of COX-2 inhibitors declined, e.g., in 2005 following rofecoxib withdrawal in Australia, celecoxib prescription declined by 23%. As a result, a slight increase in paracetamol use was observed [60]. Unfortunately, neither unfavorable results of clinical trials nor the EMA precautions had any impact on trends in COX-2 utilization in Lithuania—a consistent increase in COX-2 utilization with no (even transient) decrease was observed during the study period. Cardiovascular risk associated with COX-2 is not simply theoretical as it is well known and based on high-quality evidence and resulting in highly increased risk for arterial thrombosis, including myocardial infarction and stroke [19]. It should be noted that the age-standardized death rate for ischemic heart disease in the latest available year is highest in Lithuania [61]. Therefore, the EMA precautions on COX-2 use should be especially applicable for Lithuania. Incompliance with these recommendations and negligent COX-2 utilization patterns cause real harm for patients, which can be life-threatening or even fatal.

In 2007, the EMA CHMP recommended to limit the use of nimesulide, i.e., to use the lowest effective dose for no longer than 15 days. These limits were raised in the assessment of nimesulide benefit–risk ratio for its hepatotoxicity [62]. Due to concerns about the risk of hepatotoxicity, nimesulide has been withdrawn from market in several countries (Spain, Finland, Belgium, France, and Ireland) [63]. Despite the above-mentioned restrictions on the use of nimesulide, the utilization of this medicinal product in Lithuania increased by 45.6% during 2005–2016. In 2012, the EMA reviewed a benefit–risk ratio of nimesulide again and restricted its use more strictly—nimesulide should only be used as a second choice and only in the treatment of acute pain or primary dysmenorrhea. However, nimesulide utilization continued to increase despite the second warning. The EMA CHMP has also restricted the use of piroxicam due to concerns about its serious adverse skin and GI reactions [26]. Nevertheless, the use of this NSAID did not decrease, and, contrarily, a total increase by 102.3% was observed when recommendations on safety were approved in 2007.

In 2013, the EMA Pharmacovigilance Risk Assessment Committee (PRAC) concluded that the effects of diclofenac on the heart and circulation when given systemically were similar to those of selective COX-2 inhibitors and recommended to apply the same CV precautions as for selective COX-2 inhibitors [64]; however, during these three years, diclofenac utilization did not decrease and reached the largest diclofenac utilization indicators over the last 11 years.

Naproxen and low-dose ibuprofen (<1200 mg/day) are considered to have the most favorable thrombotic cardiovascular safety profile of all NSAIDs and are typically recommended as first-line analgesics [65]. Positive trends were observed in line with ibuprofen utilization—it consistently increased in Lithuania during the study period. On the other hand, in 2015, the EMA PRAC recommended updating the advice on the use of high-dose (>2400 mg) ibuprofen because of increased risk of CV side effects [23]. This study was unable to differentiate utilization data according to the dose actually prescribed for a patient; therefore, it is very difficult to conclude if international recommendations had any impact on ibuprofen utilization trends. Contrary to positive trends of ibuprofen utilization, the utilization of naproxen remains one of the lowest among all non-opioid analgesics in Lithuania. Besides, naproxen appeared on the market only in 2011 even though marketing authorization was granted in 1996. Meanwhile, in the Scandinavian countries (e.g., Sweden and Norway), the utilization of naproxen is continuously increasing and this drug becomes one of the most popular analgesics.

### 4.4. Reasons for Noncompliance with Guidelines

This study has revealed some serious pain management-associated problems causing potential health risks such as inadequate N02B and M01A group distribution and noncompliance with international recommendations. There can be several reasons for that. Firstly, the pharmacovigilance system has not been completely developed in Lithuania. Only 476 adverse reactions were reported during 2015 in Lithuania (2,888,558 inhabitants [66]), and only 3% of them were attributed to musculoskeletal system-affecting drugs [67], while the Danish (5,681,810 inhabitants [68]) Medicines Agency received altogether 7538 adverse drug reaction reports [69] on the same year. One more example showing the ineffectiveness of the Lithuanian pharmacovigilance system could be the analysis of reports on adverse reactions from the French pharmacovigilance database over the 2002–2006 period. A total of 42,389 reports on serious adverse reactions associated with eight NSAIDs (aceclofenac, diclofenac, ketoprofen, meloxicam, naproxen, nimesulide, piroxicam, and tenoxicam) were identified [70]. It is obvious that not all adverse reactions are reported in Lithuania. Hence, there is a misconception that current patterns of NSAID use are appropriate and result in no need for change of current traditions or introducing new evidence-based guidelines in real life clinical practice. On the other hand, there is no system designated to deal with drug-related problems. In many countries, clinical pharmacologists play this role. For instance, in Denmark, Norway, and Sweden, where clinical pharmacology is well developed, the drugs of the N02B group with other analgesics and antipyretics are used more than those of M01A (NSAIDs) for pain management, while, in the Baltic countries, which lack the role of clinical pharmacologist, NSAIDs are much more popular than other analgesics, resulting in potential serious health risks. Furthermore, there are no nationally approved guidelines supporting the rational and evidence-based use of non-opioid analgesics. Currently, pharmaceutical companies and traditions have the major impact on particular non-opioid choice. Nevertheless, some measures to solve the problems mentioned above have already been initiated. Experts in the field of clinical pharmacology, neurology, and pain medicine have developed the national evidence-based recommendations on the rational use of paracetamol and NSAIDs. Regulatory authorities (the Ministry of Health and the State Medicines Control Agency) should further support the implementation of these recommendations in clinical practice. Likewise, an educational campaign to promote an appropriate use of non-opioid analgesics for both health care specialists and the general population should also be launched and implemented.

## 5. Conclusions

Based on the benefit–risk ratio and in accordance with regulatory authority recommendations and international guidelines, paracetamol, naproxen, and low-dose ibuprofen are considered analgesics of first choice. Unfortunately, neither EMA safety policy on NSAID nor the WHO pain treatment guidelines had a sufficient impact on the rational use of NSAIDs in Lithuania. The use of NSAIDs restricted by the EMA (diclofenac, COX-2 inhibitors, nimesulide, and piroxicam) remains high or even increases, while the utilization of safer alternatives (paracetamol and naproxen) remains relatively low compared with the Scandinavian countries. Incompliance with the international guidelines may result in increased morbidity, mortality and higher costs for health care. Additional complex measures such as the implementation of national guidelines and educational programs are required to change current trends in non-opioid analgesic use.
